# NAD+-Increasing Strategies to Improve Cardiometabolic Health?

**DOI:** 10.3389/fendo.2021.815565

**Published:** 2022-01-31

**Authors:** Francisco Blanco-Vaca, Noemi Rotllan, Marina Canyelles, Didac Mauricio, Joan Carles Escolà-Gil, Josep Julve

**Affiliations:** ^1^Servei de Bioquímica, Hospital de la Santa Creu i Sant Pau i Institut d’Investigació Biomèdica Sant Pau, Barcelona, Spain; ^2^ Departament de Bioquímica i Biologia Molecular, Universitat Autònoma de Barcelona, Barcelona, Spain; ^3^ CIBER de Diabetes y Enfermedades Metabólicas Asociadas (CIBERDEM), Madrid, Spain; ^4^ Institut de Recerca de l’Hospital de la Santa Creu i Sant Pau i Institut d’Investigació Biomèdica de l’Hospital de la Santa Creu i Sant Pau, Barcelona, Spain; ^5^Servei de Endocrinologia i Nutrició, Hospital de la Santa Creu i Sant Pau i Institut d’Investigació Biomèdica Sant Pau, Barcelona, Spain

**Keywords:** vitamin B3, clinical trials, obesity, diabetes mellitus, nicotinamide

## Abstract

Depleted nicotinamide adenine dinucleotide (NAD+) is a common hallmark of metabolic disorders. Therefore, NAD+-increasing strategies have evolved as a potential therapeutic venue to combat cardiometabolic diseases. Several forms of vitamin B3, i.e., nicotinamide and nicotinamide mononucleotide, and especially nicotinamide riboside, have attracted most interest as potentially safe and efficacious candidates for NAD+ restoration. Herein, we dissected the characteristics of the latest clinical trials testing the therapeutic potential of different vitamin B3 molecules to improve cardiometabolic health, with a special focus on randomized, placebo-controlled clinical trials performed in the context of obesity or other pathologies, mainly linked to cardiovascular system and skeletal muscle functionality. The favorable outcomes *via* NAD+-increasing strategies found in the different studies were quite heterogeneous. NAD+-increasing interventions improved capacity to exercise, decreased blood pressure, increased the anti-inflammatory profile and insulin-stimulated glucose disposal, and reduced the fat-free mass. Except for the decreased blood pressure, the significant results did not include many hard clinical end points, such as decreases in weight, BMI, fasting glucose, or HbA1c percentage. However, the analyzed trials were short-term interventions. Overall, the accumulated clinical data can be interpreted as moderately promising. Additional and long-term studies will be needed to directly compare the doses and duration of treatments among different vitamin B3 regimes, as well as to define the type of patients, if any, that could benefit from these treatments. In this context, a major point of advancement in delineating future clinical trials would be to identify subjects with a recognized NAD+ deficiency using novel, appropriate biomarkers. Also, confirmation of gender-specific effect of NAD+-increasing treatments would be needed.

## Introduction

Nicotinamide adenine dinucleotide (NAD+) is a dual molecule. Apart from its well-established role as a cofactor for redox reactions and, in its reduced form, as an electron donor to the mitochondrial oxidative phosphorylation system for the synthesis of ATP, NAD+ has also been described as a signaling molecule. Indeed, NAD+ is also a substrate for NAD+-consuming enzymes involved in the metabolic adaptations that govern cell metabolism and survival.

It is known that low levels of NAD+ result from altered NAD+ homoeostasis. Impaired NAD+-mediated signaling and concurrent alterations in dysfunctional mitochondria commonly underlie cardiometabolic disorders, such as type 2 diabetes, non-alcoholic fatty liver, and aging.

NAD+ precursors are naturally found in food, and their use has emerged as a strategy for NAD+ replenishment and hence to favorably influence NAD+ dependent pathways and rescue tissues from the adverse consequences of aging and metabolic diseases (1). NAD+ content in animal and human tissues can be increased *via* the supplementation of different precursors (2–4). Although NAD+ can be synthesized directly from tryptophan (*de novo* pathway), it is more efficiently generated from other precursors, such as nicotinic acid (NA) (Preiss-Handler pathway) and nicotinamide (NAM) (Salvage pathway). Nicotinamide phosphoribosyltransferase (NAMPT), which converts NAM into nicotinamide mononucleotide (NMN), is the key step for NAD+ synthesis (5). Another source of NAD+ comes from nicotinamide riboside (NR).

NAD+ precursors that are used to elevate NAD+ availability in target tissues have demonstrated efficiency in improving insulin sensitivity and reducing diabetes burden and associated metabolic derangements in preclinical models. Actually, a growing number of studies have successfully used different vitamin B3 forms to boost NAD+ production and positively influence aging and cardiometabolic diseases in experimental *in vivo* models, including our studies performed in the context of accelerated atherosclerosis or obesity (4, 6, 7). However, less consistent results have been obtained in clinical studies.

This review analyzes the accumulating evidence from the latest clinical studies that used different NAD+-increasing strategies to improve cardiometabolic health, with a special focus on randomized, double-blind, placebo-controlled clinical trials, since they are considered the “gold standard” for testing clinical intervention-based studies (8). Particularly, a Pubmed search on clinical reports published in the last 5 years on this topic using “nicotinamide” and "randomized clinical trials" as keywords was performed.

## Results of Therapeutic Interventions With Different Forms of Vitamin B3

A plethora of pathways require NAD+ as a coenzyme. The easiest strategy to increase NAD+ *in vivo* is *via* the provision of NAD+ precursors, which include NA (also termed niacin), NAM, NR, or NMN (5, 6). However, these NAD+ intermediates can exhibit a distinct behavior due to the different tissue distribution and relative abundance of enzymes or transporters involved in NAD+ metabolism.

### NA

NA has been the most widely used form of vitamin B3 in clinical practice due to its hypolipemic properties, i.e., triglyceride-reducing and high-density lipoprotein cholesterol (HDL-C)-raising effects (5). Besides such lipid effects, NA also interacts with the GPR109A receptor expressed in immune cells, thus blunting immune activation (5). Although such NA properties are beneficial to treat hyperlipidemia, NA administration is frequently associated with some serious adverse effects, i.e., flushing, deterioration of insulin resistance, and hepatic and gastrointestinal toxicity (5), which reduce medication adherence. Moreover, early reports supporting efficacy of NA in secondary cardiovascular prevention were not confirmed by more recent, large prospective trials, including 29,087 subjects, the AIM-HIGH (9), and the HPS2-THRIVE (10), which used extended-released NA and laropiprant, a selective antagonist of prostaglandin D2 receptors, to control adverse effects (5). Since NA also ameliorated wound healing and cardiac function after myocardial infarction *via* prostaglandin D2 receptor subtype 1-mediated M2 macrophage polarization, the inhibition of prostaglandin D2 signaling could have attenuated NA cardiovascular benefits (11). The latter finding would be consistent with data from earlier clinical trials using NA, showing a reduction of mortality in subjects nine years after stopping NA therapy (12). It is also possible that the protective effect of NA is no longer significant in the context of previous statin treatments (9). However, NA may also be a potential source for NAD+ synthesis (13), and could present beneficial effects raising tissue NAD+ levels in case of deficiency. In this regard, the effect of NA in 5 subjects (4 females) with adult-onset mitochondrial myopathy subjects and systemic NAD+ deficiency and 10 healthy controls (8 females) was recently assessed (14). NA (up to 750–1,000 mg/day) or placebos were administered in a non-randomized, open-able parallel assignment to subjects and their matched controls for 10 or 4 months, respectively. The NA administration resulted in blood elevations of NAD+ in all subjects, up to eightfold, also restoring the muscle NAD+ levels of the subjects (14). Noteworthy was that muscle strength and mitochondrial biogenesis increased in all subjects. Furthermore, the muscle metabolome in the subjects with adult-onset mitochondrial myopathy shifted toward that of the healthy controls, whereas liver fat accumulation decreased by 50%. Adiponectin concomitantly increased, a finding consistent with previous studies in subjects treated with a high dose of NA (i.e., 1,500 mg/day) (15, 16). Important, the blood analysis was revealed as a useful sample to identify NAD+ deficiency in subjects with myopathy (14).

### NAM

Unlike NA, NAM is not considered a GPR109A agonist, thus avoiding prostaglandin-related vasodilatory side effects. NAM is a widely available dietary supplement, which, at doses of no more than 3 g/day, has been proven to be safe (17). In the ENDIT study, 276 subjects with type 1 diabetes mellitus were administered with NAM in daily doses of 1.2 g/m^2^ (25–50 mg/kg), up to a maximum of 3 g/day for 5 years, with minimal side effects (17). However, this clinical trial with NAM failed to prevent the progression to overt of autoimmune type 1 diabetes (18). Importantly, higher doses of NAM have been reported to produce severe but reversible hepatotoxicity (19). Effects on glucose kinetics and insulin sensitivity are inconsistent, but minor degrees of insulin resistance have also been reported (17).

NAD+ deficiency is also a risk factor for acute kidney injury (20). Recently, a phase 1 placebo-controlled study of oral NAM demonstrated that a dose-related increase in circulating NAD+ was associated with less acute kidney injury (20). Similarly, although minimal, NAD+ biosynthesis from tryptophan, at the expense of the quinolinate phosphoribosyltransferase, also prevents renal NAD+ depletion and mediates resistance to acute kidney injury. Overall, these data support the concept that increasing NAD+ levels could be beneficial for the treatment of some forms of kidney disease.

Consistent experimental observations have suggested similar NAD+-increasing benefits by NAM for the treatment of heart failure with preserved ejection fraction (21). In the former study, it was found that the dietary intake of NAD+ precursors was negatively associated with decreased blood pressure and cardiovascular mortality in a 20-year follow-up of the Bruneck Study, and persisted after adjusting for caloric intake, age, BMI, sex, smoking, diabetes, alcohol intake, and categories of food items.

### NMN

The safety of NMN administration (100, 250, and 500 mg) was recently investigated in 10 healthy men, aged from 40 to 60, in a single-arm, non-randomized intervention clinical trial (22). In this study, single oral administrations of NMN did not cause any significant clinical symptoms or changes in heart rate, blood pressure, oxygen saturation, or body temperature 5 h after NMN administration. The plasma concentrations of NAM metabolites (N-methyl-2-pyridone-5-carboxamide and N-methyl-4-pyridone-5-carboxamide) were dose-dependently increased in treated subjects.

In relation to the impact of NMN intervention on dynamic metabolic adaptations, NMN supplementation enhanced aerobic capacity in amateur runners in a 6-week randomized, double-blind, placebo-controlled, four-arm clinical trial (**Table 1**) (23). In this study, up to 48 young and middle-aged recreational runners were included (40 males and 8 females, aged between 27–50 years, with a previous history of 1–5 years of regular exercise). The participants were randomized into three groups (with a ratio of 10 males to 2 females per group), taking a low (300 mg/day), medium (600 mg/day), and a high dosage group (1,200 mg/day). A control group (placebos) followed the same male to female ratio. The runners trained 5–6 times/week in sessions of 40–60 min. The participants did not show any obvious side effects. Exercise combined with NMN intake did not change the body composition or BMI, but analysis of the change from baseline over the 6-week treatment showed that the oxygen uptake (VO_2_), the percentage of maximum oxygen uptake (VO_2_ max), and the power at the first and second ventilatory thresholds increased in the medium- and high-dose NMN groups compared with the placebo.

**Table 1:** Recently (from 2018) published randomized, double-blind, placebo-controlled clinical trials that tested NMN administration on healthy individuals or in individuals with overweight and obesity.

Country, NCT code	Dose, duration of treatment, number, and main clinical characteristics	Main results	Reference
China	NMN or placebos (300, 600, and 1,200 mg) were administered during 6 weeks to 48 subjects (8 women), aged 27–50. The recreational runners, training 5–6 times a week, were divided into four groups.	No major adverse effects. Evidence of increased plasma metabolites. Medium and high doses presented increased exercise capacity (i.e., increased oxygen uptake). No effect on cardiac function, BMI, or fat-free mass.	(23)
2000035138			
USA	NMN or placebo per day (250 mg) over 10 weeks, given to 25 women with mean age of 61, BMI of 33.5 kg/m^2^, plasma glucose of 5.65 mmol/L, and HbA1c of 5.55%,	No major adverse effects. Increased insulin-stimulated glucose disposal and skeletal muscle insulin signaling. Up-regulated expression of platelet-derived growth factor receptor β and other genes related to muscle remodeling. No change in body weight or composition.	(24)
03151239			

BMI, body mass index; NMN, nicotinamide mononucleotide; NCT, clinical trial identifier (ClinicalTrials.gov).

In a very recent report (24), 25 overweight or obese women (BMI ranged from 25.3 to 39.1 kg/m^2^, mean age of 61.5) and prediabetes completed a randomized, double-blind, parallel-assigned treatment with 250 mg of NMN or placebos over 10 consecutive weeks (**Table 1**). There were no major adverse side effects. Plasma concentrations of N-methyl-2-pyridone-5-carboxamide and N-methyl-4-pyridone-5-carboxamide metabolites of NMN and NAD+ content in peripheral blood mononuclear cells (PBMC) increased in subjects with NMN supplementation, but not in those taking placebo. Neither body weight nor body composition differed on NMN treatment. In contrast, insulin-stimulated glucose disposal, as assessed by hyperinsulinemic-euglycemic clamp, and skeletal muscle insulin signaling (AKT and mTOR total protein and the phosphorylated forms) increased with NMN, thus revealing improved insulin signaling in treated subjects. In line with this, NMN also upregulated the expression of platelet-derived growth factor receptor β (which enhances insulin-stimulated AKT phosphorylation and glucose transport) and other genes in skeletal muscle that are involved in tissue remodeling. However, no changes in muscle mitochondrial oxidative capacity or muscle function were observed. NMN could therefore be a useful intervention approach in obese, prediabetic subjects, which often present muscle loss with impaired glucose metabolism (25). Further studies are warranted to clarify the potential contribution of NMN in the latter condition.

### NR

NR is also a source for NAD+ synthesis. Consistently, human blood NAD^+^ levels rose 2.7-fold in a pilot study of one healthy 52-year-old male individual, receiving an oral daily dose of NR (1,000 mg/day) for 7 consecutive days (26). More recently, the different daily doses of NR, administered orally (i.e., 100, 300, and 1,000 mg) in 12 healthy subjects (6 male and 6 female) with ages from 30–55 and BMI 18.5–29.9 kg/m^2^, were investigated in the context of a randomized, double-blind, three-arm crossover pharmacokinetic study design. The NR produced concomitant dose-dependent elevations in the blood NAD^+^ metabolome. The rise in nicotinic acid adenine dinucleotide (NAAD) was a highly sensitive biomarker of effective NAD+ replenishment (26).

In an independent, non-randomized, open-label pharmacokinetic study made in 8 healthy volunteers (6 female, 2 male, age range 21–50 years), 250 mg of NR was orally administered on days 1 and 2, and then increased to 1,000 mg twice daily on days 7 and 8. On the morning of day 9, subjects completed a 24-h pharmacokinetics study after receiving 1,000 mg of NR (27). The treatment was well tolerated with no apparent adverse side events. Significant increases from baseline to mean NR blood concentrations at a steady state were observed for both NR and NAD+, with a 100% increase in the NAD+ levels. Absolute changes from baseline to day 9 in NR and NAD+ levels were highly correlated (27).

In another study, a 2- × 6-week randomized, double-blind, placebo-controlled, crossover clinical trial, 24 healthy lean (average BMI = 24 ± 4 kg/m^2^) men (*n* = 11) and women (*n* = 13), aged 55 to 79, received 500 mg of NR twice a day (**Table 2**) (28). NR supplementation was well tolerated by all participants. The NR increased the NAD+ and related metabolite concentrations in PBMC. Importantly, treatment with NR significantly decreased blood pressure, aortic pulse wave velocity, and carotid artery compliance. There were no changes in total energy expenditure or energy expenditure from fat oxidation under resting conditions, blood glucose control, insulin sensitivity, aerobic exercise capacity, or motor function. It is worth noting that this study was conducted in non-obese, healthy middle-aged, and older adults without baseline metabolic dysfunction (28), and this could have limited the improved metabolic outcomes.

**Table 2:** Recently (from 2018) published randomized, double-blind, placebo-controlled clinical trials that tested NR administration on individuals either healthy, aged, or overweight or obese.

Country, NCT code	Dose, duration of treatment, number, and main clinical characteristics	Main results	Reference
USA, 02921659	Administered 1,000 mg per day, 6 weeks in a crossover design, *n* = 24 (13 women), described as 55–79-year-old healthy, aged, lean subjects (BMI 24 ± 4 kg/m^2^).	No major adverse effects. Increased NAD+ and related metabolites in peripheral blood mononuclear cells. Treatment decreased blood pressure, aortic pulse wave velocity, and carotid compliance. No change in total energy intake and expenditure, BMI, % body fat, glucose and insulin metabolism, or exercise capacity.	(28)
USA	Administered 100 mg, 300 mg, 1,000 mg per day, 8 weeks parallel study, 133 (85 women); 40–60 years old, healthy overweight (BMI 25–30 kg/m^2^) in a parallel study.	No major adverse effects. Dose-dependent increase of NAD+ whole blood and urine metabolome. No changes in blood pressure, mean heart rate, weight, or resting energy expenditure.	(29)
0271593			
UK, USA, Australia	Single center, double blind, placebo-controlled, and crossover study on 12 male aged volunteers, recruited from the Birmingham 1,000 Elders group (https://www.birmingham.ac.uk/research/activity/mds/centres/healthy-ageing/elders.aspx), age 70–80 years, BMI 20–30 kg/m^2^ receiving 1,000 mg of NR or placebos during 21 days	Muscle RNA sequencing revealed that NR down-regulated energy metabolism and mitochondria pathways without altering mitochondrial bioenergetics or whole-body glucose homeostasis, decreasing circulatory cytokines (especially IL-6, IL-5 and IL-2). NR did not alter hand-grip strength.	(30)
02950441			
Denmark, 2303483	2,000 mg per day, 12 weeks: 40 men, 40–70-year-old, insulin resistant, with mean BMI of 38.5 kg/m^2^, glucose of 5.6 mmol/L and HbA1c of 5.7%.	No major adverse effects. No changes in carbohydrate metabolism, resting energy expenditure, lipolysis, lipid oxidation, or body composition. No impact on glucose tolerance, β-cell secretory capacity, α-cell function or incretin secretion, or bile acid levels. Moreover, there were no changes in NAD+ metabolite concentration in skeletal muscle.	(32–34)
Netherlands	1,000 mg per day, 6 weeks, 13 (7 women), age 59 ± 5, healthy overweight or obese (BMI 30.2 ± 2.6 kg/m^2^)	No major adverse effects. Increased markers of NAD+ synthesis in skeletal muscle. Minor increase in fat free mass, especially in women (6 out of 7). Minor increase in sleeping metabolic rate that could be related to the change in fat free mass. Acetylcarnitine concentrations in skeletal muscle and the capacity to form acetylcarnitine upon exercise were increased in NR in respect to placebo. No effects of NR were found on insulin sensitivity, mitochondrial function, hepatic and intramyocellular lipid accumulation, cardiac energy status, cardiac ejection fraction, ambulatory blood pressure, plasma markers of inflammation, or energy metabolism.	(37)
02835664			

BMI, body mass index; NAD, nicotinamide adenine dinucleotide; NR, nicotinamide riboside; NCT, clinical trial identifier (ClinicalTrials.gov).

The effect of different doses of NR (100, 300, and 1,000 mg/day) on NAD+ metabolite concentration in urine and blood was investigated in 133 healthy males and females (54%–66%, depending on the group), aged 40–60, and overweight (BMI 25–30 kg/m^2^), in an independent 8-week randomized, double-blind, placebo-controlled parallel clinical trial (29). The NR increased, dose-dependently and significantly, whole blood NAD+ (from 22% to 142%) and other NAD+ metabolites within the first 2 weeks. The NAD+ elevations were maintained thereafter, without any adverse effects (29).

In another study, 12 healthy old men, aged from 70 to 80, and BMI 20–30 kg/m^2^ (able to discontinue aspirin for 3 days prior to a muscle biopsy, statins and vitamin D one week before the beginning of the study) were supplemented with 500 mg of NR, twice a day, for 21 days, in a placebo-controlled, randomized, double-blind, crossover trial (**Table 2**) (30). NR supplementation decreased levels of circulating inflammatory cytokines (30) but did not produce favorable changes in body weight, blood pressure, lipid profile, fasting glucose and insulin, or homeostatic model assessment of insulin resistance (HOMA-IR). Although the supplementation of NR elevated the muscle NAD+ metabolome, it did not influence mitochondrial bioenergetics or whole-body glucose homeostasis. Despite the hand-grip strength values in these subjects being consistent with muscle aging, targeted NAD+ metabolome analysis likely revealed NAD+ sufficiency. Overall, these findings suggest that chronological age *per se* may not be a major factor impairing NAD+ metabolism.

In an early phase I study of 5 patients admitted with a class IV New York Heart Failure Classification to demonstrate NR safety and feasibility, blood samples were obtained before and after 5–9 days of oral NR administration (NR was up-titrated over 3 days to a final NR dose of 1,000 mg twice daily) (31). The treatment enhanced PBMC respiration and reduced pro-inflammatory cytokine gene expression in 4 male subjects, as one of the subjects did not complete the study.

Several clinical trials have focused on searching for the beneficial effects of NR in individuals with overweight or obesity. Data from the first of these clinical trials were published in three different reports (32–34). This clinical trial consisted of a 12-week randomized, double-blind, placebo-controlled, parallel-group trial conducted in 40 non-smoking, medication-free middle-aged males (40–70 years) with obesity (BMI mean of 32.85 kg/m^2^), sedentarism (< 30 min of daily exercise), with a mean fasting glucose of 5.6 mmol/L and HbA1c of 5.7% (**Table 2**) (32). They were administered NR at 1,000 mg, twice a day (*n* = 20) or a placebo (*n* = 20). After 12 weeks of treatment, increased concentrations of NAD-derived metabolites were detected in the urine of NR-treated subjects, thereby showing that the oral NR was readily absorbed, metabolized, and excreted. No serious adverse events were observed upon NR supplementation, and the safety blood tests were normal. HbA1c, insulin sensitivity, endogenous glucose production, glucose disposal and oxidation, resting energy expenditure, lipolysis, oxidation of lipids, and body composition did not change with NR supplementation (32). The NR supplementation did not affect fasting, the post-glucose challenge concentrations of glucose, insulin, C-peptide, glucagon, glucagon-like peptide-1 or gastric inhibitor polypeptide, or the beta-cell function (as revealed by the oral glucose test tolerance testing) (33). Protein levels of NAMPT in skeletal muscle decreased by 14% with NR, while NAD+ levels, as well as gene expression and protein abundance of other NAD+ biosynthetic enzymes, remained unchanged between the groups. The respiratory capacity of skeletal muscle mitochondria, abundance of mitochondrial associated proteins, mitochondrial fractional area, or network morphology in the skeletal muscle of NR-treated participants did not differ from the placebo group (34). Overall, these data do not support the hypothesis that dietary NR supplementation has a significant impact on skeletal muscle mitochondria in obese and insulin-resistant men. This could at least partly be explained by the fact that, despite having elevated urine levels of NAD+-derived metabolites, the tissue content of NAD+, NADH, NADP+, or NADPH in the skeletal muscle in men receiving NR did not differ from those receiving placebos (35, 36).

In another randomized, double-blinded, placebo-controlled, crossover intervention study, 13 healthy overweight or obese men and women (age: 59 ± 5; BMI: 30.2 ± 2.6; *n* = 7 women) receiving either NR (1,000 mg/day) or placebo supplementation during 6 consecutive weeks were followed by broad metabolic phenotyping, including hyperinsulinemic-euglycemic clamps, magnetic resonance spectroscopy, muscle biopsies, *ex vivo* mitochondrial function, and *in vivo* energy metabolism assessment (**Table 2**) (37). NR supplementation resulted in elevations of NAD+ synthesis, such as NAAD and methyl-nicotinamide (me-NAM), in skeletal muscle. In particular, the percentage of fat-free mass (62.65% ± 2.49% compared with 61.32% ± 2.58%), the skeletal muscle acetylcarnitine (4,558 ± 749 compared with 3,025 ± 316 pmol/mg dry weight), and the capacity to form acylcarnitine upon exercise were increased upon NR supplementation (37). To our knowledge, such an effect of NR on body composition in humans has not been reported before. Consistently, the sleeping metabolic rate was also affected by NR supplementation. The NR-treated subjects also showed concomitantly higher metabolic rates than the placebo subjects. In contrast, insulin sensitivity was not improved in NR-treated men. However, NR did increase the fat-free mass in 6 out of 7 women, whereas such an increase was only observed in 1 out of 6 men; thus, there might be a gender-specific response pattern in the effect of NR supplementation.

## Discussion

Thanks to the extensive research already performed with some vitamin B3 forms, its considerable safety has been confirmed. The analyzed clinical trials were built on this concept. However, special consideration should be given to the fact that some enzymes determining NAD+ levels have been related to cancer progression, at least at an experimental level (38).

Data from different clinical trials are somewhat difficult to compare and interpret, mainly due to differences in the vitamin B3 forms, doses, duration of treatment, gender, and cardiometabolic characteristics of the studied subjects.

One of the emerging interpretations is that gender could be a determinant of positive response to treatment, since most reports that obtained significant cardiometabolic differences in vitamin B3 treatment comprised a higher proportion of women (24, 28, 37) than those in which no such differences were found (32–34). However, this was not without exceptions. Elhassaen et al. (30) found some differences in men only, and Conze et al. (2019) did not find such differences, even though the study featured a majority of women (29). Interestingly, the percentage of men in the AIM-HIGH (9) and HPS-THRIVE (10) trials was around 85%.

Apart from gender-specific differences, cardiometabolic status may also differ among studies. In this regard, subjects with obesity were excluded from these two latter studies (29, 30), whereas the analysis of the effect of NR was conducted in subjects with obesity and prediabetes in other clinical trials (24, 37). In the study of Remie et al. in 2020, basically only the women in the study showed a response to the NR (37). In contrast, the main difference in the clinical trials that failed to find response to vitamin B3 treatment was directed only to men (32–34), even though it should also be considered that it also used twice the dose of the other studies. Furthermore, circadian rhythms influence the action of at least some of the key enzymes involved in the bioavailability of the currently used NAD+ precursors, and this could also be considered to be minimizing the variability among future clinical trials (39). Last, in the clinical trial of Dollerup et al. (32–34) there were some indications of limited NR tissue bioavailability.

As mentioned, the favorable outcomes in response to NAD+ precursor supplementation found in the different studies were quite heterogeneous. Thus, NAD+-increasing interventions increased capacity to exercise (23), decreased blood pressure (28), decreased anti-inflammatory circulating cytokines (30, 31), increased insulin-stimulated glucose disposal (24), or decreased fat-free mass (37). Most of these concrete findings were not reproduced in the rest of the clinical trials. Most of the positive findings were not very hard clinical end points (except the decreased blood pressure). No significant reductions in body weight or its surrogate, BMI, fasting glucose, or %HbA1c were observed in response to NAD+ precursors. This could be due to the short duration of the clinical trials analyzed, which obviously impacted the probability of the change in HbA1c percentage.

The health potential of targeting NAD+ homeostasis is still an underexplored field for future interventions of non-communicable disorders. Given the contribution of NAD+-sirtuin signaling on mitochondrial and metabolic homeostasis, an interesting proposal for future clinical trials could be the study of the effects of vitamin B3 interventions in combination with moderate exercise (40) to directly assess the metabolic adaptations provided by restored NAD+ homeostasis, concurrent with improved mitochondrial function. Moreover, future clinical trials should focus on subjects with selective biomarkers of recognized NAD+ deficiency (26) and analyzing the gender-effect in order to maximize the chances of success.

## Conclusion

Although NAD+-increasing strategies have the potential to improve overweight, obesity, and cardiometabolic health *in vivo*, additional larger and long-term studies are needed to shed light on its precise clinical indications, preferred vitamin B3 form, doses, and treatment duration.

## Author Contributions

Conceptualization: FB-V, NR, and JJ. Writing-Original Draft Preparation: FB-V and JJ. Writing-Review and Editing: MC, DM, and JE-G. Funding Acquisition: FB-V, NR, JE-G, and JJ. All authors contributed to the article and approved the submitted version.

## Funding

This work was partly funded by the Instituto de Salud Carlos III and FEDER “Una manera de hacer Europa” grant PI19/00136 (JE-G), PI18/00164 (to FB-V), and PI17/00232 (JJ). This work was also funded by Ministerio de Ciencia, Innovación y Universidades (PID2019-104367RB-100), as well as from the Agencia Estatal de Investigación (AEI/10.13039/501100011033) within the Subprograma Ramón y Cajal (RYC-201722879) to NR. JJ was recipient of a Miguel Servet Type 2 contract (CPII18/00004; ISCIII). CIBERDEM is an Instituto de Salud Carlos III project. JE-G and JJ are members of Red de Investigación en “Enfermedades Metabólicas y Cáncer” (RED2018-102799-T), Ministerio de Economía y Competitividad (MINECO), Madrid, Spain. Institut de Recerca de l’Hospital de la Santa Creu i Sant Pau is accredited by the Generalitat de Catalunya as Centre de Recerca de Catalunya (CERCA).

## Conflict of Interest

The authors declare that the research was conducted in the absence of any commercial or financial relationships that could be construed as a potential conflict of interest.

## Publisher’s Note

All claims expressed in this article are solely those of the authors and do not necessarily represent those of their affiliated organizations, or those of the publisher, the editors and the reviewers. Any product that may be evaluated in this article, or claim that may be made by its manufacturer, is not guaranteed or endorsed by the publisher.
